# A Digital Twin-Driven Life Prediction Method of Lithium-Ion Batteries Based on Adaptive Model Evolution

**DOI:** 10.3390/ma15093331

**Published:** 2022-05-06

**Authors:** Dezhen Yang, Yidan Cui, Quan Xia, Fusheng Jiang, Yi Ren, Bo Sun, Qiang Feng, Zili Wang, Chao Yang

**Affiliations:** 1 School of Reliability and Systems Engineering, Beihang University, Beijing 100191, China; dezhenyang@buaa.edu.cn (D.Y.); cuiyidan@buaa.edu.cn (Y.C.); jiangfusheng@buaa.edu.cn (F.J.); renyi@buaa.edu.cn (Y.R.); sunbo@buaa.edu.cn (B.S.); fengqiang@buaa.edu.cn (Q.F.); wzl@buaa.edu.cn (Z.W.); 2 School of Aeronautic Science and Engineering, Beihang University, Beijing 100191, China; yangchao@buaa.edu.cn

**Keywords:** life prediction, reliability evaluation, lithium-ion battery, digital twin, model evolution, predictive maintenance

## Abstract

Accurate life prediction and reliability evaluation of lithium-ion batteries are of great significance for predictive maintenance. In the whole life cycle of a battery, the accurate description of the dynamic and stochastic characteristics of life has always been a key problem. In this paper, the concept of the digital twin is introduced, and a digital twin for reliability based on remaining useful cycle life prediction is proposed for lithium-ion batteries. The capacity degradation model, stochastic degradation model, life prediction, and reliability evaluation model are established to describe the randomness of battery degradation and the dispersion of the life of multiple cells. Based on the Bayesian algorithm, an adaptive evolution method for the model of the digital twin is proposed to improve prediction accuracy, followed by experimental verification. Finally, the life prediction, reliability evaluation, and predictive maintenance of the battery based on the digital twin are implemented. The results show the digital twin for reliability has good accuracy in the whole life cycle. The error can be controlled at about 5% with the adaptive evolution algorithm. For battery L1 and L6 in this case, predictive maintenance costs are expected to decrease by 62.0% and 52.5%, respectively.

## 1. Introduction

As a new energy resource, lithium-ion batteries have been widely used in mobile phones, laptops, energy storage systems, military equipment, aerospace, etc. [1,2] With the rapid development of industrial technology and the continuous improvement of the degree of product integration and intelligence, the application scenarios of lithium-ion batteries tend to be complicated. The predictive maintenance of lithium batteries can effectively reduce maintenance costs, shorten the failure time, and improve system reliability. Predictive maintenance is strongly based on the health state of the battery; therefore, accurate life prediction is the key to effective predictive maintenance.

Lithium-ion batteries’ life prediction and reliability evaluation are the key issues in engineering applications [3,4]. The existing methods mainly include data-based, model-based, and data–model fusion [1,2,3,4,5,6,7,8]. Li [9] proposed a prognostic framework shared by multiple batteries with a variant long short-term memory (LSTM) neural network (NN) method to improve the accuracy of health state estimation and life prediction. A deep learning-based stacked denoising autoencoder method is proposed to predict battery life by Xu et al. [10]. Xie [11] analyzed the growth of the SEI layer based on a pseudo-two-dimensional (P2D) electrochemical model, and constructed the degradation model of batteries. Moreover, a multi-parameter coupling degradation model was established to predict the calendar and cycle life of batteries by He [12]. Ren [13] proposed a reliability life assessment method of the lithium-ion battery pack based on the multiphysical coupling model. Then, the multiphysical behavior and life in different situations were analyzed and predicted. Ma [14] combined the capacity degradation data and open-circuit-voltage parametric model to improve the accuracy of capacity estimation.

At present, the life prediction for lithium-ion batteries is mainly based on the prior knowledge of historical data and models, which may not meet the needs of accurate predictive maintenance. Life prediction should be developed from merely prior knowledge to real-time and historical information integration. With the development of sensor technology and data analysis methods, the concept of the digital twin provides inspiration and technical ways to solve the above problems [15]. A digital twin uses digital technology and virtual model simulation technology to explore and predict the operating state of physical space, which provides the important theoretical basis and technical support for the connection and real-time interaction between virtual and physical space [16,17]. The digital twin can be naturally linked with the reliability requirements of products for its main advantage of real-time and accurate mapping between virtual and physical space. The literature shows that digital twin technology has been applied to evaluate and predict the performance degradation, failure, and life of products, for example, the concept of digital twins was applied to the health monitoring, life prediction, and maintenance support decision-making of aircraft by the U.S. Air Force Research Laboratory [18,19,20] and the General Electric Global Research Center [21]. The performance and reliability of underwater unmanned vehicle systems were predicted and improved based on digital twin technology by Demetrious et al. [22]. Rajesh [23] applied digital twin technology into the life prediction of automobile brake pads. A wear prediction model of tool cutting using digital twin technology was proposed by Dary et al. [24]. A digital twin application framework and a method of structural health state detection and residual life prediction for aircraft were established based on digital twins by Beihang University [25,26,27,28]. Digital twin technology has been well applied in life prediction and health assessment of aircraft, automobiles, and mechanical products.

In the application of lithium batteries, Monika et al. [29] proposed a cloud battery management system based on the concept of a digital twin to solve the problem of limited data storage in vehicle battery management systems. A hybrid twin model for predicting the degradation of lithium-ion batteries under real driving conditions was proposed by San Carlos et al. [30]. Park [31] constructed a digital twin model of an all-solid-state battery from physical and electrochemical perspectives to predict the electrochemical behavior of the battery. There are also scholars who study models and methods related to digital twins in the field of supercapacitor materials [32,33]. A digital twin based on the performance degradation assessment model of the battery was established by Qu [34] to estimate the capacity under dynamic operating conditions. Moreover, a framework of a digital twin for the modeling and fault diagnosis of the battery was proposed by Billy et al. [35]. The existing research and applications of digital twin technology for batteries mainly focus on the technical framework and the construction of deterministic models of electrochemical or degradation behavior. In practice, because of inevitable differences in the production process, connection conditions, and operating conditions, the degradation process of the battery life cycle has the characteristics of multistate, dynamic, and random uncertainty [36,37]. Thus, how to accurately describe the dynamic evolution and random uncertainty characteristics of the lithium battery model is the key to realizing accurate life prediction and digital twinning. However, the existing research about the digital twin of batteries lacks consideration of uncertainty and model evolution to the best of our knowledge.

To solve the above problems and realize an accurate online life prediction and reliability evaluation, a digital twin for the reliability of lithium-ion batteries is proposed in this paper based on the stochastic degradation model and a Bayesian-based adaptive evolution method. The paper is organized into six sections. The framework of a digital twin for reliability of lithium-ion batteries is proposed in Section 2. The digital twin models for reliability are established in Section 3. The model parameters are summarized, and the experimental verification is described in Section 4. Several cases of life prediction, reliability evaluation, and predictive maintenance are analyzed in Section 5. The conclusions and future work are presented in Section 6.

## 2. The Framework of Digital Twin-Driven Life Prediction of Lithium-Ion Batteries

**Figure 1:**
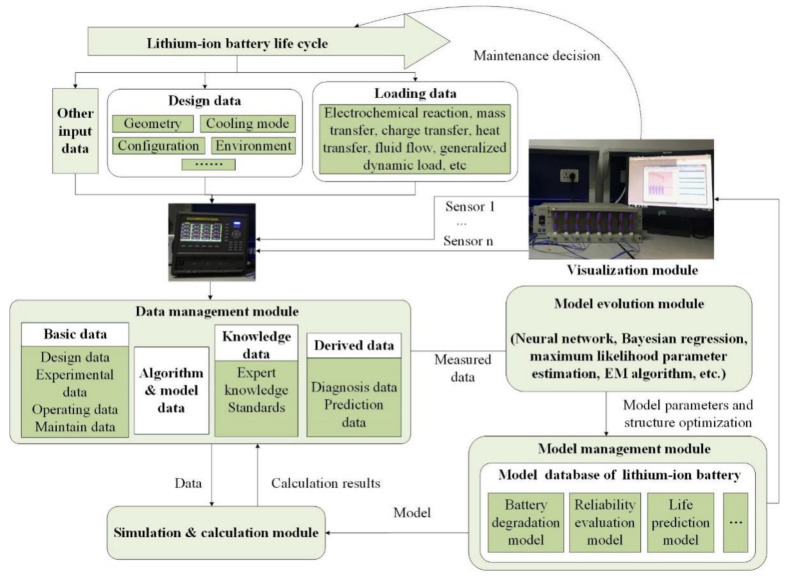
The framework of the digital twin for the reliability of lithium-ion batteries.

According to the framework of digital twins [25,26,27], the digital twin for the reliability of lithium-ion batteries is established with its structure shown in Figure 1, which is mainly composed of the data acquisition module, data management module, model management module, simulation and calculation module, model evolution module, visualization module, etc.

(1) Data acquisition module

To realize the interaction between the digital twin and physical entities, battery information needs to be collected by data acquisition, including the design data, operation data, real-time feedback data, etc. The function of the data acquisition module is data collection and transmission in the whole life cycle of the battery.

(2) Data management module

The data management module is used to process and manage all kinds of data related to the digital twin, including the basic data composed of design data, experimental data, operating data and maintenance data; the algorithm and model data; expert knowledge and standards data; and the derived data composed of diagnosis and prediction results.

(3) Model management module

In the model management module, all models can be stored, used, managed, and updated. The digital twin model for the reliability of batteries contains the degradation model, the reliability evaluation model, the life prediction model, etc.

(4) Simulation and calculation module

Various algorithms are integrated into the simulation and calculation module. This module will interact with the data and model management module to realize the function of description, prediction, diagnosis, analysis, and evaluation of the battery.

(5) Model evolution module

With the use of lithium-ion battery products, the digital twin will continuously accumulate a large amount of data during the whole life cycle including mission profile, working environment, sensor acquisition, and maintenance measured data. Combining the historical and real-time data, the structure and parameters of the digital twin models can be updated and evolved by the model evolution method, such as neural networks, Bayesian regression, maximum likelihood parameter estimation, EM algorithm, etc. This model evolution module will constantly improve the accuracy of the digital twin mapping to the actual battery.

(6) Visualization module

In the visualization module, according to the user’s need, the digital twin models, historical data, real-time data, prediction, diagnosis, and evaluation results of batteries can be visualized by visual design, programming, and the set up human–computer interaction interface.

(7) Other functional modules

In addition to the main modules mentioned above, the digital twin for the reliability of batteries also includes the maintenance decisions, implementation, feedback, etc. For maintenance decisions, the fault prediction will be implemented in the digital twin by using historical data and real-time data. It will then provide the corresponding maintenance strategies according to the failure mode and severity.

## 3. Digital Twin-Driven Life Prediction Model for Lithium-Ion Batteries

Based on the proposed framework of a digital twin for the reliability of lithium-ion batteries, a digital twin model for reliability based on the remaining useful life cycle prediction is established to verify the feasibility of the method, including stochastic degradation model, life prediction model, and the Bayesian-based evolution model.

### 3.1. Stochastic Degradation Model

(1) Degradation model

Chemical and mechanical degradation are generally considered to be the main mechanisms causing battery degradation [38]. There are many reasons for capacity fading [39,40], such as the formation, cracking, and dissolution of the solid electrolyte interface (SEI), electrolyte decomposition, and lithium plating. To facilitate the construction and verification of the digital twin model for the reliability of lithium-ion batteries, the following assumptions are made:

(1) The mechanical degradation caused by fatigue, cracking, and structural changes related to discharge and charging rates is ignored. Chemical degradation is considered to be the main reason for the loss of active lithium ions [38,41].
(2) During the cycling of lithium-ion batteries, temperature is a key factor affecting battery capacity degradation [41,42]. It has been considered that the current indirectly affects the degradation of the battery in the form of heat generation and temperature rise [43,44]. Thus, the capacity decay of the battery can be expressed by the Arrhenius formula [41]:

(1) $$\frac{dC_{fade,N}\left(t\right)}{dt}=A_{d}\exp\left[-\frac{E_{a}}{RT\left(t\right)}\right]$$

(2) $$C_{fade,N}=\int_{0}^{t_{N}}A_{d}\exp\left[-\frac{E_{a}}{RT\left(t\right)}\right]dt$$

where *C_fade, N_* is the accumulated capacity fade of the battery in the *N*th cycle (mAh). *A_d_* and *E_a_* represent the concentration degradation rate (mAh·s^−1^) and activation energy (J·mol^−1^), respectively. R is the ideal gas constant (8.314 J mol^−1^·K^−1^). *T*(*t*) is the time-dependence average battery temperature (K). *t_N_* represents the end time of the discharge process (s).

The suitable working temperature for lithium-ion batteries is usually about 298.15–318.15 K [45,46,47]. Therefore, in actual engineering, a battery thermal management system (BTMS) is usually applied to improve the operating environment [48,49]. Thus, it is assumed that the battery system operates in a good temperature range, and the degradation model of Equations (1) and (2) is suitable for the temperature range of 298.15–318.15 K.

(2) Stochastic model

Due to the differences in the production process and operating environment in practice, the degradation of lithium-ion batteries has a certain degree of randomness and dispersion. For the nonlinear and fluctuating characteristics of the degradation process, randomness is defined to describe the stochastic degradation during the life cycle of a battery. In addition, for the differences of multiple battery degradation, the dispersion is defined to describe the stochastic degradation characteristic of the same type or batch of batteries. According to the normal distribution and Weibull distribution [37], a stochastic degradation model is established with the probability density function (PDF) expressions listed as follows:

(3) $$\mathrm{Normal}\;\mathrm{distribution}:\;f_{fade}\left(x,\mu ,\sigma \right)=\frac{1}{\sqrt{2\pi }\cdot \sigma }\cdot e^{-\frac{{\left(x-\mu \right)}^{2}}{2\sigma^{2}}}$$

(4) $$\mathrm{Weibull}\;\mathrm{distribution}:\;f_{fade}\left(x,\beta ,\eta \right)=\frac{\beta }{\eta }\cdot {⟮\frac{x}{\eta }⟯}^{\beta -1}\cdot e^{-{⟮\frac{x}{\eta }⟯}^{\beta }}$$

where *μ* and *σ* are the location parameter and scale parameter of normal distribution, respectively. *β* and *η* are the shape parameter and scale parameter of the Weibull distribution. The calculation formulae for mean value are shown as follows:

(5) $$D_{norm}=\mu =C_{fade}\left(T,t,...\right)$$

(6) $$D_{Weibull}=\eta \Gamma \left(1+1/\beta \right)=C_{fade}$$

For the normal distribution, the accumulated capacity fade *C_fade_* is the mean value of the distribution, whose parameters can be used to describe the stochastic degradation of the battery shown as follows:

(7) $$\mu_{fade}=C_{fade}$$

(8) $$\sigma_{fade}=\kappa \mu_{fade}$$

where *κ* is the relative co-efficient of variation (*κ =* *σ*/*μ*), which is obtained by data fitting. For the Weibull distribution, the shape parameter *β* will not change with increases in the number of cycles. Thus, the relationship between shape parameter *β* and temperature can be established by polynomial fitting. The scale parameter *η* can be derived from Equation (6). The capacity stochastic degradation model *f_ξ_*(*x*,*μ*,*σ*) that obeys the Weibull distribution is shown as follows:

(9) $$\beta \left(T\right)=f\left(T,T^{2},T^{3}\cdots \right)$$

(10) $$\eta \left(T,t\right)=\frac{\xi \cdot t}{\Gamma ⟮1+\frac{1}{\beta \left(T\right)}⟯}$$

where *ξ* is the battery degradation rate. The parameters of the above stochastic model can be obtained by fitting the degradation data of the battery.

### 3.2. Life Prediction and Reliability Evaluation Model

Based on the degradation model, the life of the lithium-ion battery can be predicted by analyzing the historical degradation trajectory and determining the failure criterion. In this paper, the remaining capacity of 80% is used as the failure criterion, and the battery capacity degradation *C_fade_* can be calculated by Equation (1). The remaining useful life (RUL) of a lithium-ion battery with the same working conditions can be calculated by the following equation:

(11) $$RUL=\frac{\left(0.2C_{nom}-C_{fade}\right)N}{C_{fade}}$$

where *C_nom_* is the nominal capacity, and *N* is the number of cycles that the battery has experienced. For temperature that changes dynamically, the remaining life can be expressed as follow:

(12) $$RUL=\frac{0.2C_{nom}-C_{fade}}{\int A_{d}\exp\left[-\frac{E_{a}}{RT\left(t\right)}\right]dt}$$

Reliability evaluation of lithium-ion batteries can be implemented by applying the stochastic degradation model. Given that the mean and standard deviation of the random variable *C_fade_* are *μ_fade_* and *σ_fade_*, and the number of samples is *n*, the upper and lower limits of the capacity degradation *CI_fade_* can be calculated by the following equations:

(13) $$CI_{fade}=\mu_{fade}\pm Z_{\alpha /2}\cdot \left(\sigma /\sqrt{n}\right),\;n\geq 30$$

(14) $$CI_{fade}=\mu_{fade}\pm t_{\alpha /2}\cdot \left(\sigma /\sqrt{n}\right),\;n<30$$

where *α* is the confidence level, and 0 < *α* < 1, *Z_α_*_/2_ and *t_α_*_/2_ are obtained according to the Z-critical and t-critical value, respectively. Based on the upper and lower limits of capacity degradation, the interval of RUL can be calculated by Equations (11) or (12).

### 3.3. Bayesian-Based Adaptive Evolution Method for Battery Models

**Figure 2:**
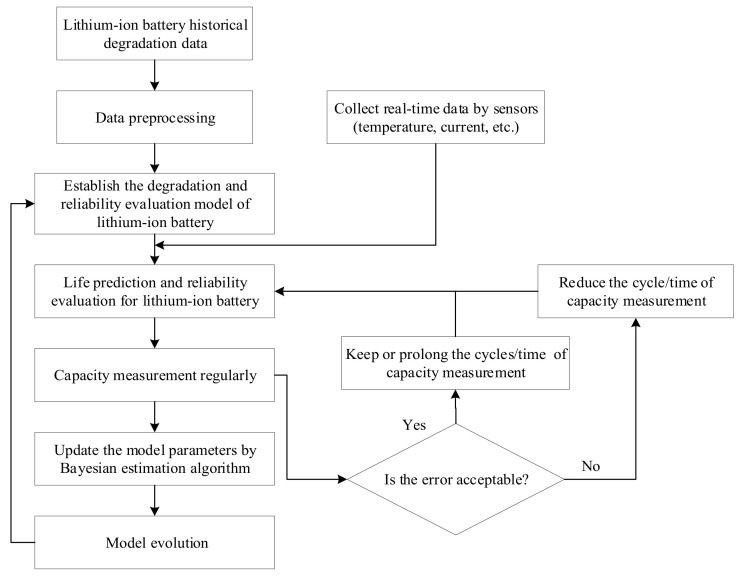
Flow chart of the Bayesian-based evolution method for the digital twin model.

The mission profiles and environmental loads of batteries varies from individual to individual, which will make the degradation trajectory and model different. Therefore, the battery model needs to be continuously revised according to the measured data to improve the prediction ability of degradation and reliability. In practice, lithium-ion battery capacity can be estimated indirectly or measured regularly, then transferred to the digital twin. The models and parameters of the digital twin will be updated by using the model evolution method. According to Refs. [50,51], the Bayesian model shows good performance in the description of dynamic characteristics. Therefore, a Bayesian-based adaptive evolution model is established for the digital twin as shown in Figure 2.

(1) Data preprocessing

Due to the influence of technological level, connection conditions, environment, and other factors, there will be some points with large deviation in the battery degradation data, these are outliers. The outliers will reduce the accuracy of the prediction model and should be eliminated. The Letts criterion method is used to identify the outliers, the method description is as follows:

In a row of measurement results with equal precision, if the absolute value of residual *V_i_* corresponding to the measurement value *X_i_* meets |*V_i_*|_max_ > 3*σ_x_*, the value *X_i_* is an outlier, which should be removed. The calculation equation of residual and standard deviation is as follows: $V_{i}=X_{i}-\bar{X}$, $\sigma =\sqrt{\sum_{i=1}^{n}V_{i}^{2}/(n-1)}$. In this paper, the capacity fade per cycle is taken as the measured value, and *X* is the average value of the capacity fade from 1 to *i* cycle.

(2) Adaptive evolution method

In the proposed adaptive evolution method, the cycle of model evolution will be dynamically adjusted according to the error between the predicted and measured results. The initial cycle of model evolution is *n*, and the acceptable range of error is determined to be *E_r_*%. If the error is within the acceptable range, the evolution cycle will be maintained or extended to *n* + *n*/2. Otherwise, the evolution cycle should be reduced to *n*/2.

(3) Bayesian estimation algorithm

To continuously estimate the parameters of the accumulated usage data of lithium-ion batteries, the Bayesian algorithm is introduced. If the historical degradation data of lithium batteries obey the normal distribution *N* (*μ*_0_, *σ*_0_^2^), the measured usage data obey the normal distribution *N* (*μ*, *σ*^2^). The Bayesian estimation algorithm for normal distribution is described as follows: 

When *σ*^2^ is known and the conjugate prior distribution of *μ* is the normal distribution *N* (*μ*_0_, *σ*_0_^2^), the posterior distribution of *μ* can be deduced by the following equation:

(15) $$\mu |\;\bar{x}\sim N\left(a,b^{2}\right),a=\frac{\frac{1}{\sigma_{0}^{2}}\mu_{0}+\frac{n}{\sigma^{2}}\bar{x}}{\frac{1}{\sigma_{0}^{2}}+\frac{n}{\sigma^{2}}},\frac{1}{b^{2}}=\frac{1}{\sigma_{0}^{2}}+\frac{n}{\sigma^{2}}$$

When *μ* is known, the conjugate prior distribution of *σ*^2^ is the inverse gamma distribution *IGa*(*α*, *β*), then $S_{\mu }^{2}$ is the sufficient statistic, and the posterior distribution of *σ*^2^ can be deduced by the following equation:

(16) $$\begin{array}{c} h\left(\sigma^{2}|\;s_{\mu }^{2}\right)\propto {\left(\sigma^{2}\right)}^{-⟮\alpha +\frac{n}{2}+1⟯}e^{\frac{\beta +\frac{3\mu }{2}}{\sigma^{2}}}\ldots \end{array}$$

(17) $$\overset{\wedge }{\sigma^{2}}=\frac{2\beta +s_{\mu }^{2}}{2\alpha +n-2}$$

When *μ* and *σ*^2^ are both unknown and independent, the Monte Carlo method can be used to estimate the parameters.

## 4. Experiment and Model Verification

### 4.1. Experimental Setup and Design

**Figure 3:**
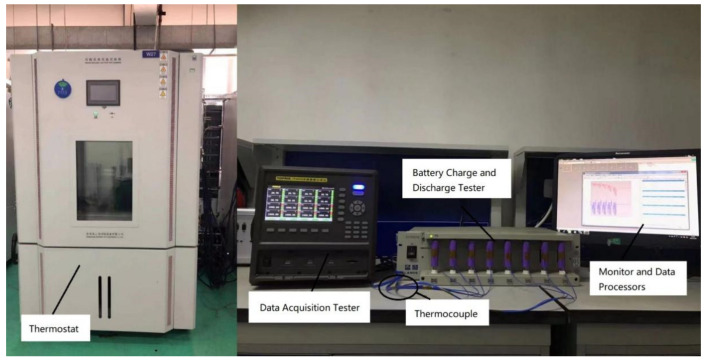
Experimental setup.

To verify the digital twin model for the reliability of lithium-ion batteries, a battery experimental platform is constructed with its structure shown in Figure 3.

(1) LAND battery test system was adopted as the charge and discharge device. The technical specifications are as follows: current range of 1 mA–5000 mA, current accuracy of 0.1% RD ± 0.1% F.S, voltage range of 2 V–15 V, voltage accuracy of 0.1% RD ± 0.1% F.S;
(2) TOPRIE TP9000 was used as the battery data acquisition system with measurement accuracy of ±0.2% F.S;
(3) A ZX GDJS was used as a thermostat with a temperature fluctuation range of ±0.5 K, and temperature homogeneity of ±2 K;
(4) K-type thermocouples were used to collect the temperature with the range of 173 K~1645 K, and measurement accuracy of ±0.05% rdg, ±0.6 K;
(5) The monitoring system consists of a computer and LAND battery network/local integrated testing software. The device specifications are Intel(R) Core (TM) i5-10210U CPU @ 1.60 GHz 2.11 GHz.

To study the randomness and dispersion of degradation, 18 batteries from the same batch of 18,650 lithium iron phosphate batteries, with nominal voltages of 3.2 V and nominal capacity of 1400 mAh, were used for capacity degradation tests. The anode, cathode, and electrolyte material are graphite, LiFePO4 and EC-EMC (3:7) solvent with LiPF6. The 18 battery cells are equally divided into three experimental groups, named L, M, and H. The labels are (L1~L6), (M1~M6) and (H1~H6), respectively. The experimental design scheme is shown in Table 1, where the temperature collection point is the center point of the side surface of the cylindrical battery.

### 4.2. Experimental Results and Analysis

**Figure 4:**
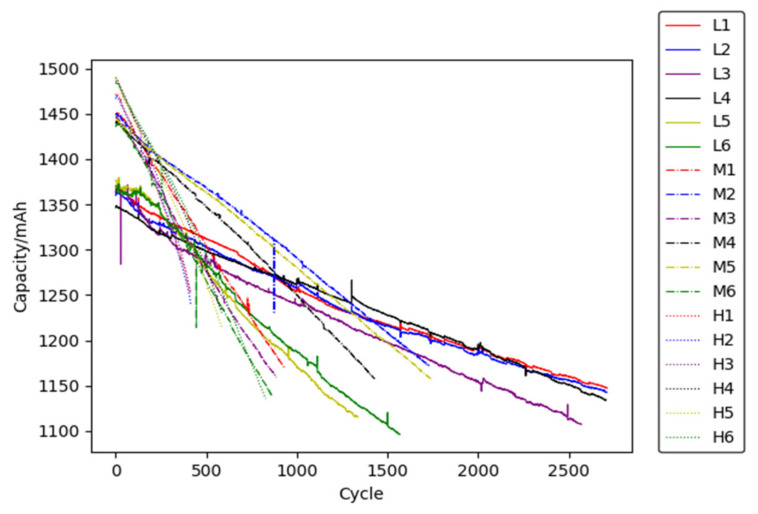
Experimental result of capacity degradation.

**Figure 5:**
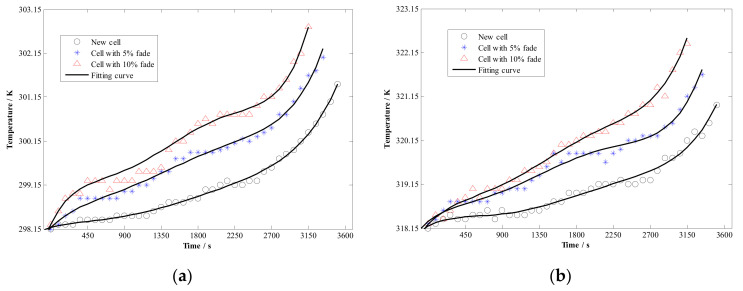
Experimental result of battery temperature; (**a**) ambient temperature 298.15 K; (**b**) ambient temperature 318.15 K.

According to the above experimental scheme, the capacity degradation test has been carried out. A total of 18 sets of the battery capacity degradation data are plotted in Figure 4, and some temperature results are shown in Figure 5.

It can be seen from Figure 4 that the battery capacity degrades rapidly in a higher ambient temperature. This is because the reaction equilibrium inside the battery will be broken through at a higher temperature, which results in the acceleration of side reaction rate and loss of active substances. It is worth noting that the battery capacity degradation curve jumps regularly. The regular jump phenomena occur at the periodical test node. It is probably because the battery recovers its reversible capacity by resting for a relatively long time at the periodical test node. This phenomenon is more pronounced at a lower temperature (298.15 K), which may be due to the slower degradation rate and the easier recovery of reversible capacity.

In addition, comparing the initial discharge capacity of the batteries at the ambient temperatures of 298.15 K, 318.15 K, and 333.15 K, it is found that the higher the ambient temperature, the greater the discharge capacity. This is because, within a certain temperature range, a higher temperature will stimulate Li+ activity, accelerate the chemical reaction rate and the transmission speed of electrolytes, improve the chemical reaction efficiency of active substances and increase the battery capacity.

From Figure 5, it can be found that the temperature rise gradually increases under the same working conditions with the degradation of the battery, which indicates that the degradation will lead to the increase of heat production during the discharge of the battery.

### 4.3. Model Verification

(1) Verification of degradation model

To verify the accuracy of the degradation model, the model parameters were obtained by fitting the battery capacity degradation data at ambient temperatures of 298.15 K, 318.15 K, and 333.15 K. The corresponding modeling and coding were implemented with Python 3.9.8. 

**Figure 6:**
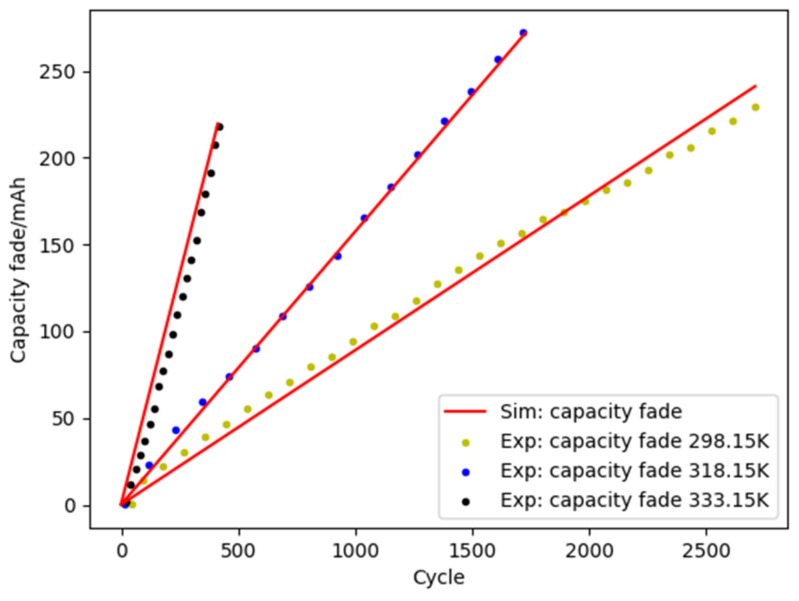
Comparison of fitting and experimental results.

The degradation data of L1, M1, and H1 batteries are used in this case. According to Equation (2), the obtained model parameters *E_a_* and *A_d_* are 27628.4 J·mol^−1^ and 1.818 mAh·s^−1^, respectively. The battery temperature *T*(*t*) in Equation (2) is fitted by the polynomial method. By substituting *E_a_* and *A_d_* into Equation (2), the fitting results of the degradation model are shown in Figure 6. The results show that the fitted curve is in good agreement with the experimental results of lithium-ion battery capacity degradation.

(2) Verification of the stochastic model

**Figure 7:**
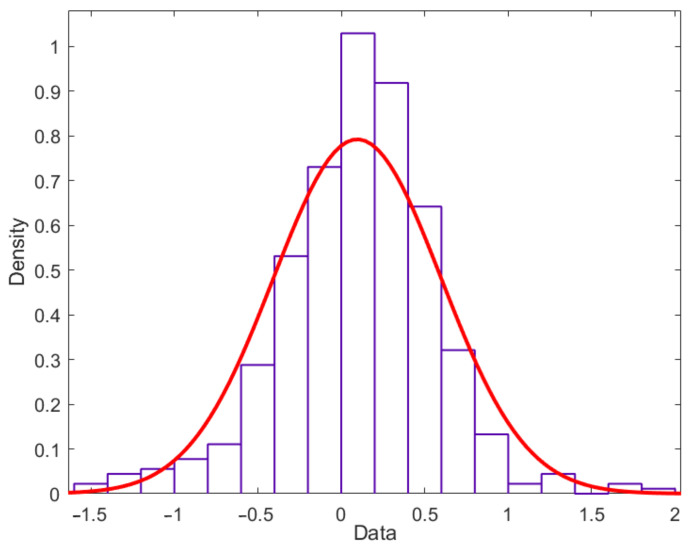
The normal distribution for L1 battery capacity degradation data.

For randomness, the normal distribution model is taken as an example. The stochastic model parameters of the L1 battery are calculated according to Equations (7) and (8) with a mean value of 0.08 and a variance of 0.57, denoted as *N* (0.08, 0.57), and the co-efficient of variation *κ* = 7.125. The normal distribution of L1 battery capacity degradation data is tested by the Kolmogorov–Smirnov test method and shown in Figure 7. The probability of degradation data obeying normal distribution is 0.943, much higher than the critical value of 0.05. Thus, the degradation data of the L1 battery can be considered to obey normal distribution.

For dispersion, the Weibull distribution model is taken as an example. If battery capacity degradation is considered as a linear process, the corresponding linear degradation function can be obtained by linearly data fitting. The slope of the function is the degradation rate *ξ*, which can be obtained by the least square fitting shown in Table 2.

**Figure 8:**
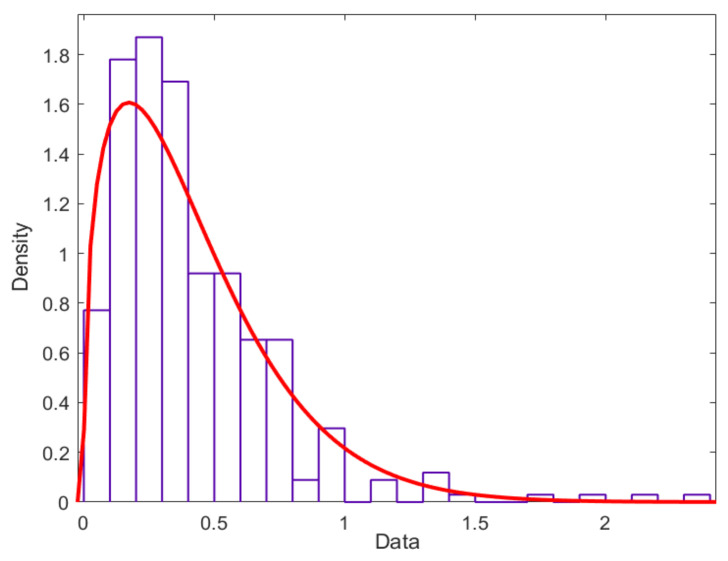
The Weibull distribution for the battery capacity degradation rate data.

Subsequently, the parameters of the Weibull distribution are obtained by fitting the capacity degradation rate data, with the results shown in Table 3 and Figure 8. The probability of the degradation rate data obeying the Weibull distribution is 0.865, much higher than the critical value of 0.05. Thus, these six sets of degradation rate data can be considered to obey the Weibull distribution.

**Table 3:** The parameters of the Weibull distribution.

Group Number	Weibull Distribution	
	*β*	*η*
L	11.3380	0.0868
M	14.6119	0.1842
H	10.6606	0.5101

According to Equations (9) and (10), the parameters of the stochastic degradation model that describe the degradation dispersion are calculated as follows:

(18) $$\beta \left(T\right)=0.7374T-55.4245$$

(19) $$\eta \left(T,t\right)=\frac{\xi \cdot t}{\Gamma ⟮1+\frac{1}{\beta \left(T\right)}⟯}$$

## 5. Case Study and Analysis

### 5.1. Life Prediction of Lithium-Ion Batteries Based on the Digital Twin

Based on the above digital twin for the reliability of lithium-ion batteries, combining the temperature information collected by the sensor, the life prediction, and reliability evaluation has been performed, including remaining cycle life prediction and the randomness and dispersion of capacity degradation. In this case, the experimental data of the L1 battery was used, including temperature, capacity degradation, and operating conditions.

(1) Cycle life prediction

**Figure 9:**
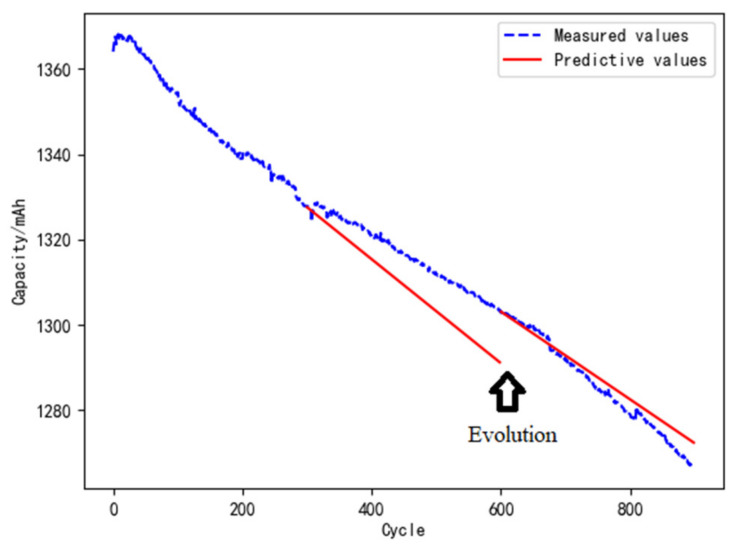
Evolution of the digital twin model for the reliability of batteries.

The data of the first 300 cycles is used as a priori knowledge after eliminating the outliers. The parameters of the degradation model are then fitted, corresponding to *E_a_* = 17468.12 J·mol^−1^, and *A_d_*= 0.039 mAh·s^−1^. The temperature data is considered as the real-time data collected by the sensor (part of which is shown in Figure 5), which is the input of the degradation model. According to the framework and method of the digital twin, the life of the L1 battery can be predicted. Before model evolution, the predicted and measured values of the remaining battery capacity of the 596th to 600th cycles are shown in Table 4. Combining the measured value of the 596th to 600th cycles, the digital twin model for reliability is evolved by using the Bayesian-based evolution method, as shown in Figure 9. After model evolution, the predicted and measured values of the 1196th to 1200th cycles are shown in Table 5.

**Table 4:** The predictive and measured value of L1 battery capacity.

Cycle Period	Initial Model					Mean Error
	596	597	598	599	600	
Predictive value (mAh)	1291.7	1291.578	1291.456	1291.334	1291.212	12.054
Measured value (mAh)	1303.59	1303.59	1303.79	1303.38	1303.2	

According to Equation (12), the remaining cycle life of the L1 battery is predicted by using the evolved digital twin model. After 900 cycles, the L1 battery operates under the ambient temperature of 298.15 K, the charge and discharge rate of 1 C-rate, and the discharge depth of 100%. The remaining cycle life is expected to be about 1846 cycles.

(2) Randomness of degradation

**Figure 10:**
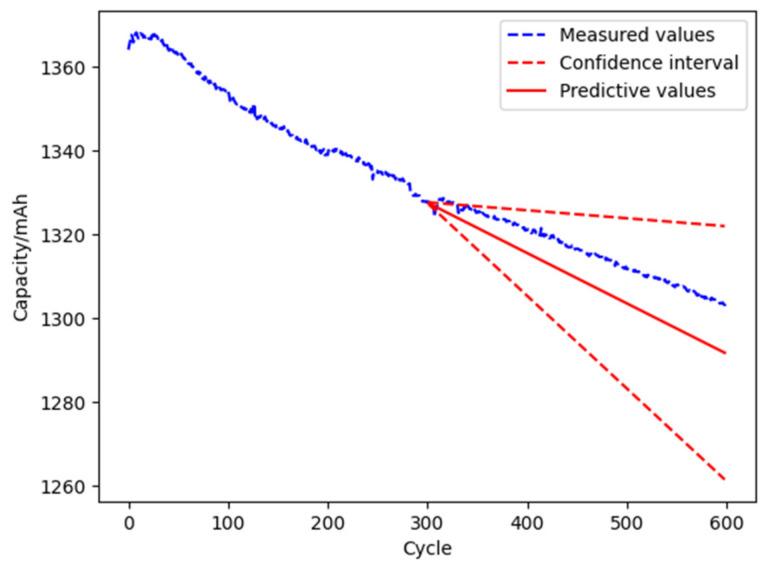
The prediction result of the randomness of battery degradation.

According to the stochastic degradation model, the historical degradation data of the first 300 cycles of the L1 battery is fitted. The result shows that the data obey the normal distribution *N*(0.12, 0.891), and the confidence interval is [0.019, 0.221] with the confidence of 95% of the mean value. The confidence interval of the L1 battery capacity degradation is deduced as shown in Figure 10. The upper limit of the predicted remaining life is about 3397 cycles, and the lower limit is about 1040 cycles. The real degradation trajectory of the L1 battery is within the confidence interval, which means that the stochastic model of the normal distribution can better describe the randomness of the capacity degradation process of a single battery.

(3) Dispersion of multi-cell degradation

In engineering, the lithium-ion battery cells need to be used in groups to meet power demand. The same type and batch of battery cells with higher consistency are usually used to ensure the overall performance of the battery pack. However, the inconsistency of degradation cannot be avoided due to differences in the production process, connection, installation, and manufacturing level. Moreover, it is uneconomical and unrealistic to obtain the operating parameters of all battery cells in practical applications. Thus, the dispersion of multiple battery cell degradation can be used to evaluate the degradation of the battery pack.

The data of the first 300 cycles of L1~L6 batteries are selected as historical data for dispersion analysis. The parameters of the degradation model are obtained corresponding to *E_a_*= 13205.57 J·mol^−1^, and *A_d_* = 0.0078 mAh·s^−1^. The capacity degradation rates of L1~L6 batteries are calculated as shown in Table 6. According to the stochastic degradation model, the dispersion of the capacity degradation rate is fitted and obeys the Weibull distribution (*β*, *η*) of *W*(4.497, 0.171).

**Figure 11:**
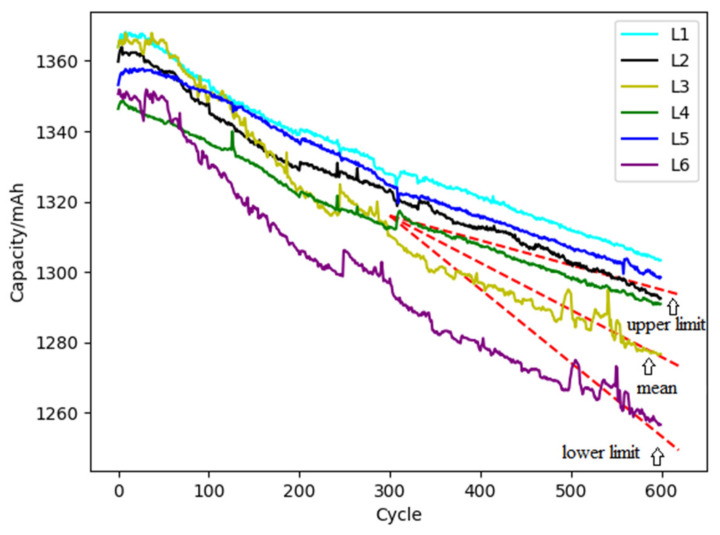
The prediction result of the dispersion of multiple battery degradation.

According to the above stochastic degradation model for dispersion, the prediction for the degradation of the battery pack is carried out with the results shown in Figure 11. The results show that the degradation of the L6 is the fastest, which may be caused by multiple stochastic factors. It means the Weibull distribution is more suitable to describe the randomness of degradation. In given load conditions of the ambient temperature of 298.15 K, the charge and discharge rate of 1 C-rate and the discharge depth of 100%, the mean value, upper limit, and lower limit of the RUL of the cells inside the battery pack are obtained according to Equation (12), which are 1496, 2146, and 1294 cycles, respectively.

### 5.2. Analysis of the Model Evolution Cycle

Real-time and accurate mapping between the physical and digital space is the key characteristic of the digital twin. The accuracy of its digital twin model for the reliability of the battery directly determines the accuracy of life prediction and reliability evaluation. The model evolution cycle will affect the accuracy of the evolved model. To analyze the influence of the model evolution cycle, the strategies of model evolution with the fixed and adaptive cycle were analyzed in this case.

(1) Model evolution with fixed cycle

**Figure 12:**
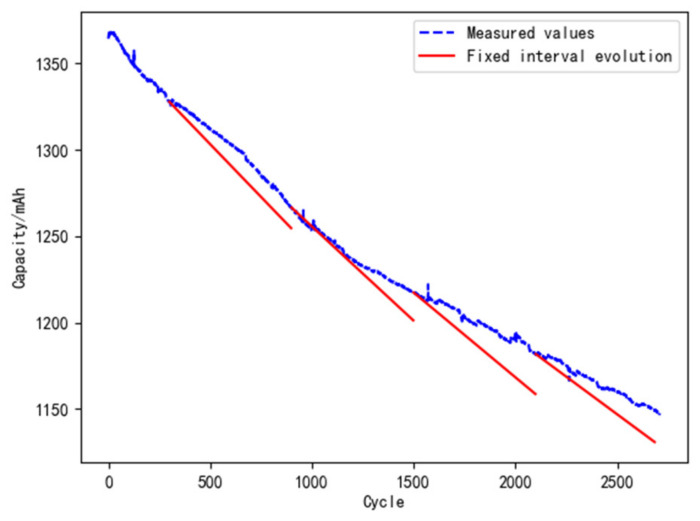
Model evolution with fixed cycles.

The strategy of 600 fixed cycles is used to update the digital twin model for reliability. The data of the L1 battery is selected for analysis, and the result is shown in Figure 12. In the life cycle of L1, the model evolves 3 times. The errors between the predicted and the measured value at the evolution points are shown in Table 7. The error equation is as follows:

(20) $$E=\frac{\left|C_{m}-C_{f}\right|}{C_{fade}}\cdot 100\%$$

where *C_m_* and *C_f_* are the measured value and predicted value of capacity, respectively.

It can be seen from Table 7 that the errors in the whole life cycle are about 11%–12% when the model is evolved with 600 fixed cycles. This means that the error cannot be reduced by regular model evolution, and it is not conductive to improving the accuracy of the digital twin.

(2) Model evolution with adaptive cycles

**Figure 13:**
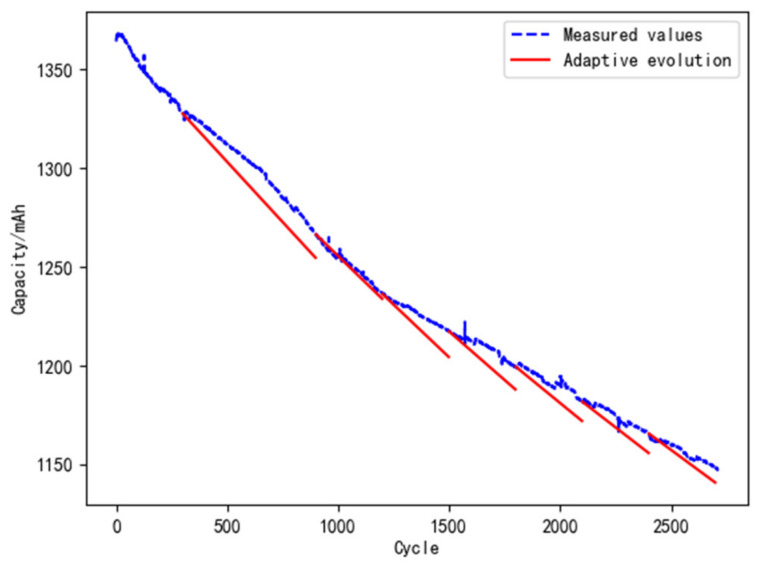
Model evolution with adaptive cycles.

The initial evolution cycle and the acceptable range of error are assumed as 600 cycles and 10%, respectively. The analysis results are shown in Figure 13.

It can be seen from Table 8 that the error between the predicted and the measured value shows a decreasing trend with the adaptive evolution algorithm, about 2% to 8%. Comparing the results of the fixed and the adaptive evolution shows that the proposed adaptive evolution method is better than the evolution method with a fixed cycle.

(3) Life prediction and model evolution based on open-source datasets

**Figure 14:**
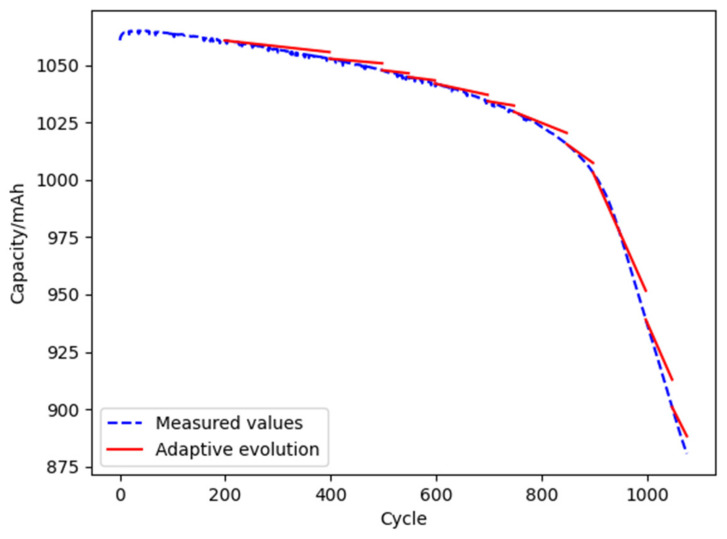
Evolution of the digital twin model based on open-source datasets.

The data of the first 200 cycles of battery batch 8_CH1 is selected to validate the digital twin model according to the open-source datasets [5]. The initial evolution cycle and the acceptable range of error are assumed as 200 cycles and 10%, respectively. The analysis results are shown in Figure 14. The errors between the predicted and the measured value at the evolution points are shown in Table 9.

It can be seen from Table 9 that the error between the predicted and the measured value shows a decreasing trend with the adaptive evolution algorithm, about 7% to 10%.

### 5.3. Analysis of Digital Twin-Based Predictive Maintenance Decision

The capacity of the lithium-ion battery system will continuously decline during use. It is assumed that the system cannot meet the power supply requirements when the remaining capacity is less than 80% of the initial capacity, which can be regarded as a failure. The predictive maintenance of a lithium-ion battery system can improve the reliability of the system in the whole life cycle. In this paper, the digital twin for reliability is applied to guide the predictive maintenance of the lithium-ion battery. The contrastive analysis of the cost of operation and maintenance with and without digital twin is implemented below.

Without a digital twin for reliability, the model will not evolve in the whole life cycle. The history degradation data of the battery is usually used to construct the average degradation model for prediction. The degradation data of L1–L6 and M1–M6 batteries are used in this case. According to the above data, the model parameters are obtained corresponding to *E_a_* = 30273.4 J·mol^−1^ and *A_d_* = 5.479 mAh·s^−1^.

Based on the digital twin for reliability, the L1 and L6 batteries are taken as two typical examples for comparative analysis. The digital twin models are evolved by the adaptive method with 600 initial cycles. The remaining cycle life of the L1 and L6 batteries is predicted under the conditions of an ambient temperature of 298.15 K, charge and discharge rate of 1 C-rate, and discharge depth of 100%. The results are shown below.

(1) Excessive maintenance

**Figure 15:**
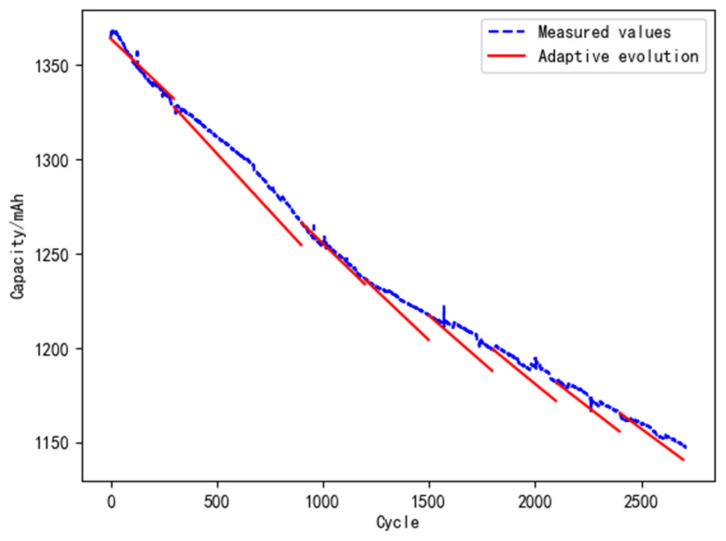
The results of model evolution of L1 batteries.

The life prediction and maintenance cost analysis results of the L1 battery are shown in Table 10, and the results of model evolution based on a digital twin are shown in Figure 15.

**Table 10:** Life prediction and maintenance cost analysis of L1 batteries.

Scheme	Predicted Value (Cycle)	Actual Value (Cycle)	Maintenance Cost
Without digital twin	2615	2878	263 *V*
Digital twin-based	2778	2878	100 *V*

It can be seen from Table 10 that the predicted life of the L1 battery with and without a digital twin is 2778 and 2615 cycles, respectively. It shows that the predicted life based on the historical data is shorter than the actual life of the L1 battery of 2878 cycles. The predictive maintenance of the L1 battery based on the predicted life will cause cost waste. Assuming that the battery usage cost per cycle is *V*, the maintenance cost of digital twin-based and without a digital twin is 100 *V* and 263 *V*, respectively.

(2) Insufficient maintenance

**Figure 16:**
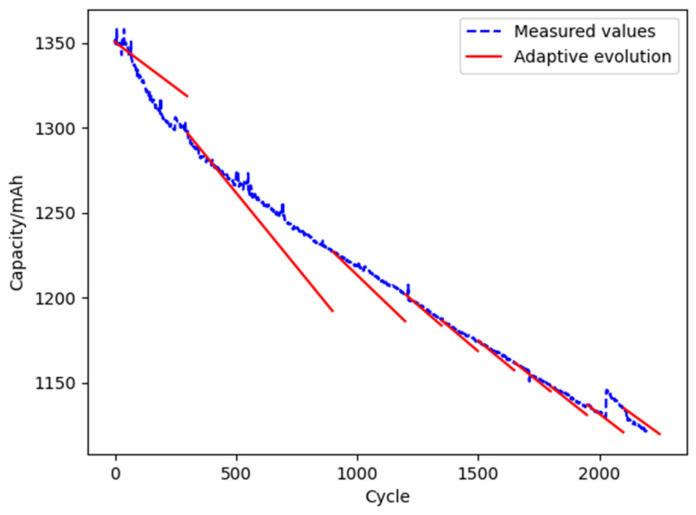
The results of model evolution of L6 batteries.

The life prediction and maintenance cost analysis of the L6 battery are shown in Table 11, and the results of model evolution based on a digital twin are shown in Figure 16.

**Table 11:** Life prediction and maintenance cost analysis of L6 batteries.

Scheme	Predicted Value (Cycle)	Actual Value (Cycle)	Maintenance Cost
Without digital twin	2615	2200	415 *P*
Digital twin-based	2397	2200	197 *P*

It can be seen from Table 11 that the predicted life of the L6 battery with and without a digital twin is 2397 and 2615 cycles, respectively. It shows that the predicted life based on the historical data is longer than the actual life of the L6 battery of 2200 cycles. The predictive maintenance of the L6 battery based on the predicted life will cause a system failure. Assuming that the economic loss per cycle is *P*, the maintenance cost of digital twin-based and without a digital twin is 197 *P* and 415 *P,* respectively.

Based on the digital twin for reliability, the maintenance cost of the L1 and L6 batteries have costs reduced by 62.0% and 52.5%, respectively. Thus, digital twin-based predictive maintenance is more economical.

## 6. Conclusions

In this paper, a framework for the use of digital twins for the reliability of lithium-ion batteries based on remaining useful cycle life prediction was proposed. The digital twin model was constructed by establishing the stochastic degradation model, the life prediction model, the reliability evaluation model, and the Bayesian-based adaptive evolution method for the model. The degradation experiments of several cells were carried out to verify the accuracy of the models. Finally, the life prediction and reliability evaluation based on digital twins has been conducted, followed by the analysis of the evolution cycle and predictive maintenance decision. The following conclusions are obtained:

(1) Based on the concept of a digital twin, the data collected by sensors can be fully utilized to evolve the models. Considering the randomness in engineering, the life prediction, and reliability evaluation can be more accurate.
(2) A Bayesian-based adaptive evolution method for the model was proposed. The accuracy of lithium-ion battery life prediction and reliability evaluation is improved effectively by adaptively adjusting the evolution cycle.
(3) Based on the digital twin for reliability, the excessive and insufficient maintenance of the battery system can be prevented, and the maintenance cost can be reduced.

In practice, the real-time acquisition of data and the dynamic evolution of models are the keys to realizing digital twins for the life prediction of lithium-ion batteries. The proposed models and method in this work provide the framework, stochastic degradation model and effective evaluation method for the realization of the digital twin, which can accurately describe the dynamic evolution and random uncertainty characteristics of the battery’s whole life cycle.

Due to the limitations of the experiments, there are still further studies that need to be conducted, including the deeper utilization of physical models and processes with a digital twin, as well as the application and verification of the digital twin for reliability under complex working conditions.

## Figures and Tables

**Table 1:** Experimental scheme for capacity degradation.

Group Number	Battery Number	Experimental Conditions	Test Methods [51,52]
L	L1~L6	Ambient temperature: 298.15 K Charge and discharge rate: 1 C-rate ^a^ Depth of discharge: 100%	(a) Charge with a constant current of 1 C-rate to the cut-off voltage (3.65 V), then charge with a constant voltage of 3.65 V until the current drops to 1/20 C-rate, rest for 1 h; (b) Discharge at a constant current of 1 C-rate to the cut-off voltage (2 V), rest for 1 h; (c) Repeat the charge and discharge process 295 times and record the discharge capacity; (d) 5 times charge and discharge test for battery capacity measurement: charge with a constant current of 0.5 C-rate to the cut-off voltage (3.65 V), then charge with a constant voltage of 3.65 V until the current drops to 1/20 C-rate, rest for 1 h; discharge at a constant current of 0.5 C-rate to the cut-off voltage (2 V), rest for 1 h.
M	M1~M6	Ambient temperature: 318.15 K Charge and discharge rate: 1 C-rate Depth of discharge: 100%	
H	H1~H6	Ambient temperature: 333.15 K Charge and discharge rate: 1 C-rate Depth of discharge: 100%	

^a^ C-rate is the measurement of the charge and discharge current with respect to its nominal capacity.

**Table 2:** Battery capacity degradation rate.

Group Number	Capacity Degradation Rate *ξ* (mAh N^−1^)					
	1	2	3	4	5	6
L	0.0787	0.0776	0.0920	0.0749	0.0806	0.0951
M	0.1573	0.1978	0.1642	0.1759	0.1813	0.1902
H	0.53	0.5356	0.5506	0.4218	0.4539	0.4215

**Table 5:** The predictive and measured value of L1 battery capacity after model evolution.

Cycle Period	Evolutionary Model					Mean Error
	1196	1197	1198	1199	1200	
Predictive value (mAh)	1241.822	1241.719	1241.616	1241.513	1241.41	4.846
Measured value (mAh)	1236.89	1237.11	1236.85	1236.5	1236.5	

**Table 6:** Capacity degradation rate of L1~L6 batteries.

Number	L1	L2	L3	L4	L5	L6
Capacity degradation rate *ξ*	0.1353	0.1452	0.2055	0.125	0.1148	0.2087

**Table 7:** The result of model evolution with fixed cycles.

Evolution Times	Predictive Value (mAh)	Measured Value (mAh)	Error
The first time	1254.612	1266.67	12.38%
The second time	1201.321	1217.45	11.00%
The third time	1158.772	1181.89	12.69%

**Table 8:** The result of model evolution with adaptive cycle.

Evolution Times	Predictive Value (mAh)	Measured Value (mAh)	Evolutionary Cycle Interval	Error
The first time	1254.612	1266.67	600	12.38%
The second time	1233.861	1236.5	300	2.07%
The third time	1204.383	1217.45	300	8.91%
The fourth time	1187.96	1199.61	300	7.09%
The fifth time	1172.01	1181.89	300	5.42%
The sixth time	1155.79	1165.73	300	5.01%

**Table 9:** The result of model evolution based on open-source datasets.

Evolution Times	Predictive Value (mAh)	Measured Value (mAh)	Error
The first time	1055.74	1052.87	35.88%
The second time	1050.84	1047.83	23.08%
The third time	1046.48	1044.87	10.06%
The fourth time	1043.41	1042	7.47%
The fifth time	1037.1	1034.32	10.47%
The sixth time	1032.42	1029.69	8.76%
The seventh time	1020.49	1015.61	10.78%
The eighth time	1007.41	1003.21	7.28%
The ninth time	951.706	939.086	10.36%
The tenth time	912.936	900.914	7.52%

## Data Availability

Not applicable.

## Nomenclature

A_d_	concentration degradation rate, mAh·s^−1^
C_fade, N_	accumulated capacity fade in the Nth cycle, mAh
CI_fade_	upper and lower limits of the capacity degradation
C_nom_	nominal capacity
E_a_	activation energy, J·mol^−1^
N	number of cycles that the battery has experienced
n	number of samples
R	ideal gas constant, 8.314J mol^−1^·K^−1^
T(t)	time-dependence average battery temperature, K
t_N_	end time of the discharge process, s
V_i_	measurement value
X_i_	residual value
X	average value of the capacity fading from 1 to i cycle
α	confidence level
μ	location parameter of normal distribution
μ_fade_	mean of the random variable C_fade_
σ	scale parameter of normal distribution
σ_fade_	standard deviation of the random variable C_fade_
β	shape parameter of Weibull distribution
η	scale parameter of Weibull distribution
κ	relative co-efficient of variation
ξ	battery degradation rate
SEI	solid electrolyte interface
BTMS	battery thermal management system
PDF	probability density function
RUL	remaining useful life
